# Supplementary material for: Detection and evaluation of copy number variation using both linked-read and short-read sequencing in New Zealand dairy cattle
Source: Front Genet. 2026 Jun 11;17:1856199. doi: 10.3389/fgene.2026.1856199 (PMC13293786; doi:10.3389/fgene.2026.1856199)
Supplement: Supplementary file 1 [file Table1.docx]

Table S1. Summary of publicly available cattle CNV datasets used for comparison, including data type, sample size, breeds, analytical methods, number of detected regions, and database availability.

| **Study** | **Data type^1^** | **Number of Samples** | **Number of Breeds** | **Software/Method** | **Number of reported CNV regions** | **DGVa^2^** | **File Location** |
| --- | --- | --- | --- | --- | --- | --- | --- |
| Liu et al., (2010) | GEN | 90 | 17 | array CGH, qPCR and FISH | 1041/200 high-confidence | Yes | <https://ftp.ensembl.org/pub/release-110/variation/vcf/bos_taurus/bos_taurus_structural_variations.vcf.gz> |
| Hou et al., (2011) | GEN | 539 | 21 | PennCNV | 682 | Yes |  |
| Hou et al., (2012) | GEN | 472 | 1 | qPCR | 3346 | Yes |  |
| Bickhart et al., (2012) | WGS | 6 | 4 | WSSD pipeline, aCGH, qPCR and FISH | 1265 | Yes |  |
| Boussaha et al., (2015) | WGS | 62 | 3 | Pindel, HD genotyping (validation) | 6426/331 high-confidence | Yes |  |
| Menzi et al., (2016) | WGS | 1 | 1 | IGV | 1 | Yes |  |
| Keel et al., (2017) | WES | 175 | 20 | DELLY2, CNVnator, CN.MOPS | 1341 | Yes |  |
| Karimi et al., (2017) | GEN | 50 | 8 | QuantiSNP | 221 | Yes |  |
| Mesbah-Uddin et al., (2018) | WGS | 175 | 3 | Genome STRiP | 8480 | Yes |  |
| Lee et al., (2023) | WGS | 266 | 1 | Smoove | 13,732 | No | <https://static-content.springer.com/esm/art%3A10.1186%2Fs12864-023-09259-8/MediaObjects/12864_2023_9259_MOESM2_ESM.xlsx> |
| Bhati et al., (2023) | WGS | 183 | 5 | Smoove | 13,942 | No | https://zenodo.org/records/8274665 |
| Grant et al., (2024) | WGS | 310 | 1 | Menta and Smoove | 30,112 (Menta)  65,550 (Smove) | No | <https://static-content.springer.com/esm/art%3A10.1186%2Fs12864-024-10812-2/MediaObjects/12864_2024_10812_MOESM3_ESM.xlsx>  and  <https://static-content.springer.com/esm/art%3A10.1186%2Fs12864-024-10812-2/MediaObjects/12864_2024_10812_MOESM4_ESM.xlsx> |

**^1^**GEN: genotyping array; WGS: whole genome sequence; WES: whole exome sequence.^2^If the detected CNV regions are included in the current Database of Genomic Variants archive.
