# Supplementary figures and images for: Detection and evaluation of copy number variation using both linked-read and short-read sequencing in New Zealand dairy cattle
Source: Front Genet. 2026 Jun 11;17:1856199. doi: 10.3389/fgene.2026.1856199 (PMC13293786; doi:10.3389/fgene.2026.1856199)

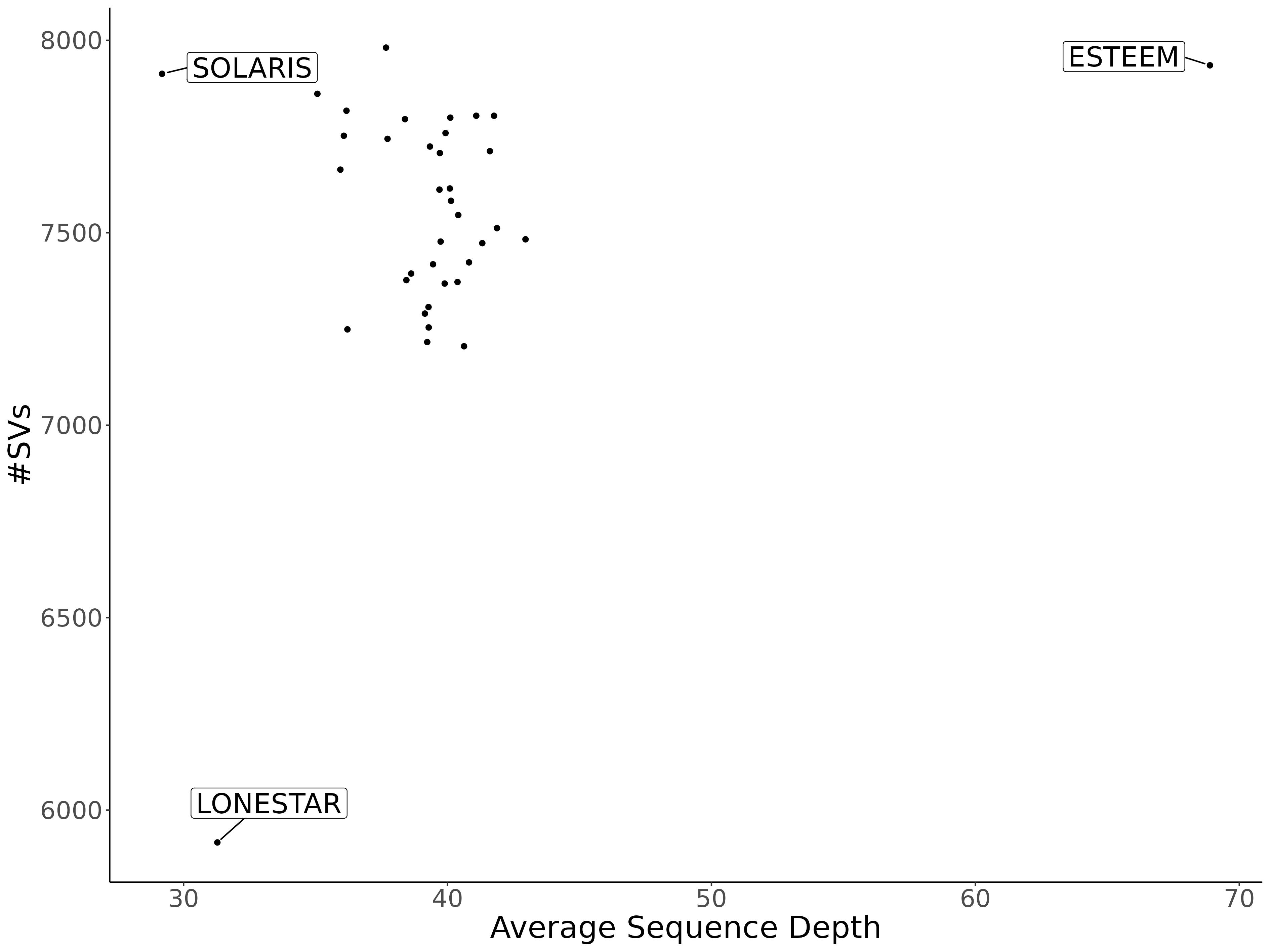

Supplement: Supplementary file 2 [file Image1.jpeg]

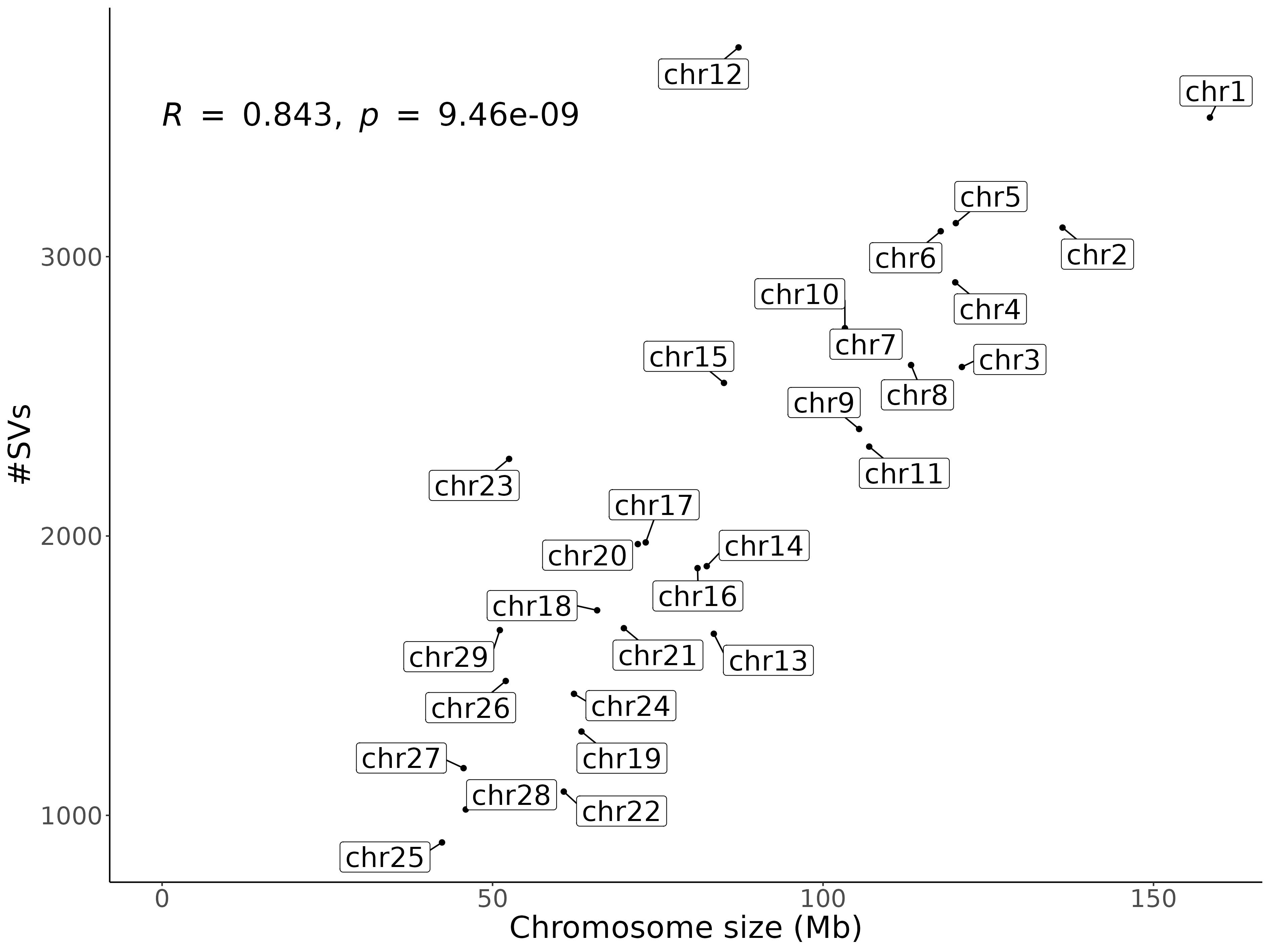

Supplement: Supplementary file 3 [file Image2.jpeg]
